# Chloroplast genome sequencing and phylogenetic analysis of *Tetrapanax papyrifer* (Hook.) K. Koch (Araliaceae)

**DOI:** 10.1080/23802359.2026.2621456

**Published:** 2026-02-03

**Authors:** Zhanwei Yu, Xianglan Liang, Song Guo, Peng Zhang

**Affiliations:** a College of Biology and Food Engineering, Guangxi Science & Technology Normal University, Laibin, PR China; b School of Chemistry and Chemical Engineering, Guangdong Pharmaceutical University, Guangzhou, PR China; c College of Smart Agriculture (College of Internet of Things Engineering), Guangxi Science & Technology Normal University, Laibin, PR China

**Keywords:** *Tetrapanax papyrifer*, chloroplast genome, molecular markers, phylogenetic analysis

## Abstract

*Tetrapanax papyrifer* (Hook.) K. Koch is an important medicinal plant in Chinese Yao medicine. In this study, the complete chloroplast genome was sequenced using high-throughput sequencing technology. Its complete chloroplast genome spans 156,165 bp in total length, comprising a large single-copy region of 86,237 bp and a small single-copy region of 17,956 bp, separated by a pair of inverted repeat regions (25,986 bp each). Phylogenetic analysis based on chloroplast genomes revealed that the genomic structure of *T. papyrifer* shows similarities with species from the genus *Heptapleurum* in the Araliaceae family. The presented chloroplast genome will facilitate further phylogenetic analyses and inform conservation strategies for this species.

## Introduction

*Tetrapanax papyrifer* (Hook.) K. Koch, 1859, a medicinal plant of the Araliaceae family, is distributed in Southwest China as well as the regions of Jiangsu, Guangdong, Hunan, Guangxi, Taiwan, and Japan (Ashida et al. 2023a, 2023b). After processing, *T. papyrifer* can be made into pith paper, pith paintings, and pith flowers (Nesbitt et al. 2010). The medicinal plant *T. papyrifer* has therapeutic functions of clearing heat and promoting diuresis, as well as regulating qi flow and promoting lactation. It is clinically indicated for treating damp-heat strangury (painful urinary disorders), edema with oliguria, and insufficient milk secretion in lactating women. Moreover, *T. papyrifer* has been recognized for its antioxidant and anti-inflammatory properties, which contribute to its widespread use as a natural therapeutic agent (Xu et al. 2025).

As a highly conserved and maternally inherited genetic system, the chloroplast genome plays a crucial role in studies of plant evolution, species identification, and phylogenetic reconstruction. Although the chloroplast genome sequence of *T. papyrifer* (GenBank accession no. MT991759) has been released in GenBank, no comprehensive study has been published to characterize its genomic structure, gene content, or evolutionary implications. Here, we assemble and annotate the complete chloroplast genome of *T. papyrifer* and perform detailed analyses of its structural features. In addition, comparative analyses with related Araliaceae species and phylogenetic reconstruction were conducted to clarify the evolutionary position of *T. papyrifer* within the family and to provide valuable genomic resources for future studies.

## Materials and methods

**Figure 1.**
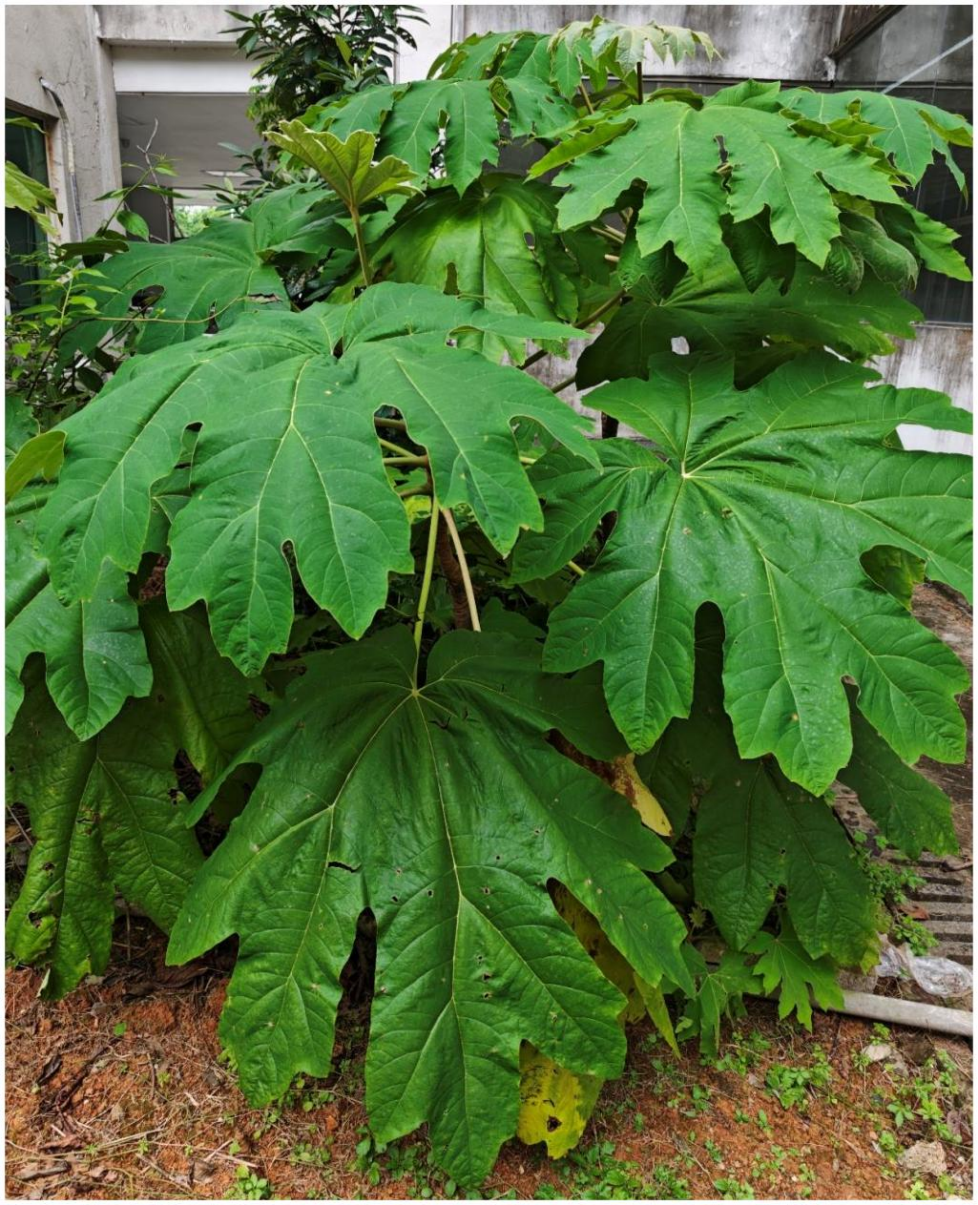
The plant depicted in the image is *Tetrapanax papyrifer*, which is characterized by large palmately lobed leaves featuring 7–12 deep lobes. The leaf margins display coarse, irregular serrations, and the leaf blade typically measures approximately 30–50 cm in diameter. The foliage possesses a thick, leathery texture, with a vibrant green upper surface and prominently elevated veins. The photo of the species was taken by the authors (Song Guo).

Fresh leaves (N: 23°46′27″, E:109°12′35″) were collected from Guangxi Science & Technology Normal University, Laibin, China. A voucher specimen was deposited in Room 404, at the College of Biology and Food Engineering, Guangxi Science & Technology Normal University (Song Guo, email: guosong0804@163.com) under the voucher number YYF20250113. Song Guo identified the plant specimen (Figure 1).

**Figure 2.**
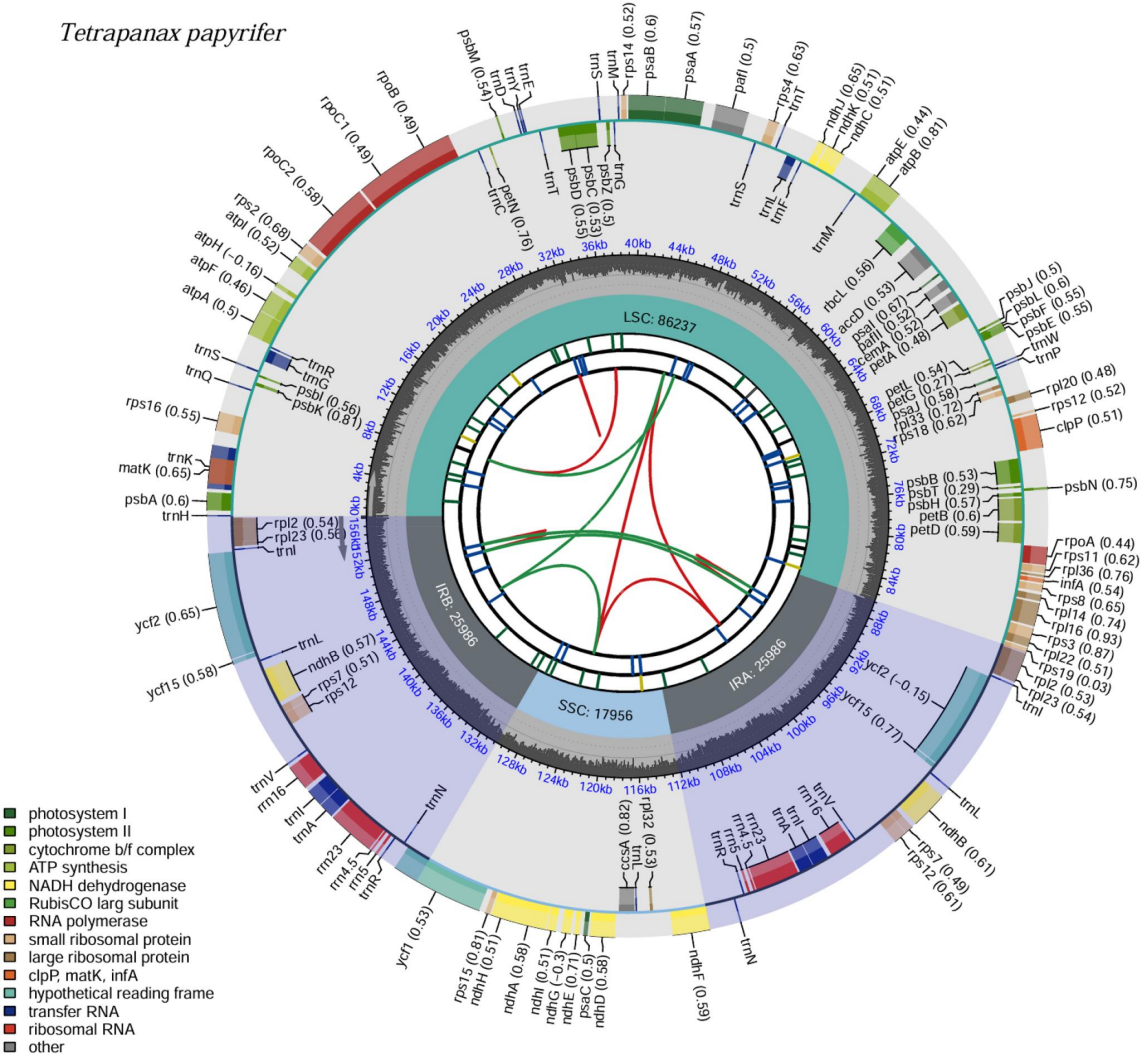
Complete chloroplast genome of *Tetrapanax papyrifer*. The map consists of six tracks, presented from the center outward: the first track depicts dispersed repeats, including direct repeats (displayed in red) and palindromic repeats (shown in green), with corresponding arcs connecting them. The second track represents long tandem repeats, illustrated as short blue bars. The third track illustrates short tandem repeats (microsatellites), presented as short bars in varying colors. The fourth track indicates the boundaries of the small single-copy (SSC), inverted repeat (IRA and IRB), and large single-copy (LSC) regions. The fifth track displays the GC content across the genome. The sixth track provides gene annotations, with optional codon usage bias noted in parentheses after each gene name. Genes from different functional categories are differentiated by color coding. Genes located inside and outside the map are transcribed in the clockwise and counterclockwise directions, respectively.

Total genomic DNA was extracted from fresh leaf tissue using the DNeasy Plant Mini Kit (Cat. No. 69104, Qiagen, Hilden, Germany). The quality and quantity of the extracted DNA were assessed, and DNA samples meeting sequencing requirements were used to construct an Illumina paired-end sequencing library (San Diego, CA) with an average insert size of 350 bp. Sequencing was performed on the Illumina platform with 150 bp paired-end reads. After sequencing, reads were mapped to the previously reported *T. papyrifer* chloroplast genome (GenBank accession no. MT991759) using BWA v 0.7.17 (Li 2013) to extract chloroplast-derived reads. These filtered reads were then assembled *de novo* using GetOrganelle v.1.7.7.1 (Jin et al. 2020) with default parameters. The assembly graph was visualized with Bandage v 0.8.1 (Wick et al. 2015) to assess the completeness of the assembled chloroplast genome. To evaluate sequencing coverage, the Illumina reads were mapped back to the assembled genome using BWA v 0.7.17 (Li 2013), and the per-base sequencing depth was calculated using Samtools v 1.13 (Danecek et al. 2021). The sequencing depth distribution across the genome was plotted using a custom Python script to verify uniform coverage. The annotation process primarily utilized existing chloroplast genomes from closely related species combined with current chloroplast genomic databases. CPGAVAS2 (Shi et al. 2019) online tools were employed to verify and refine the annotation results. Any annotation errors or inconsistencies were further checked and corrected using BLAST (Chen et al. 2015) searches and manually curated with Apollo v 1.11.8 (Lewis et al. 2002). The assembled and annotated fasta file along with GenBank file has been deposited in the NCBI GenBank database under accession number MW829284.2. To further evaluate structural differences, the IR boundary regions of our annotated genome were compared with those of the previously published genome MT991759.1 using IRscope (https://irscope.shinyapps.io/irapp/) (Amiryousefi et al. 2018). All gene features such as cis-splicing (Figure S2) and trans-splicing (Figure S3), as well as circular map (Figure 2), were visualized using CPGView (Liu et al. 2023).

**Figure 3.**
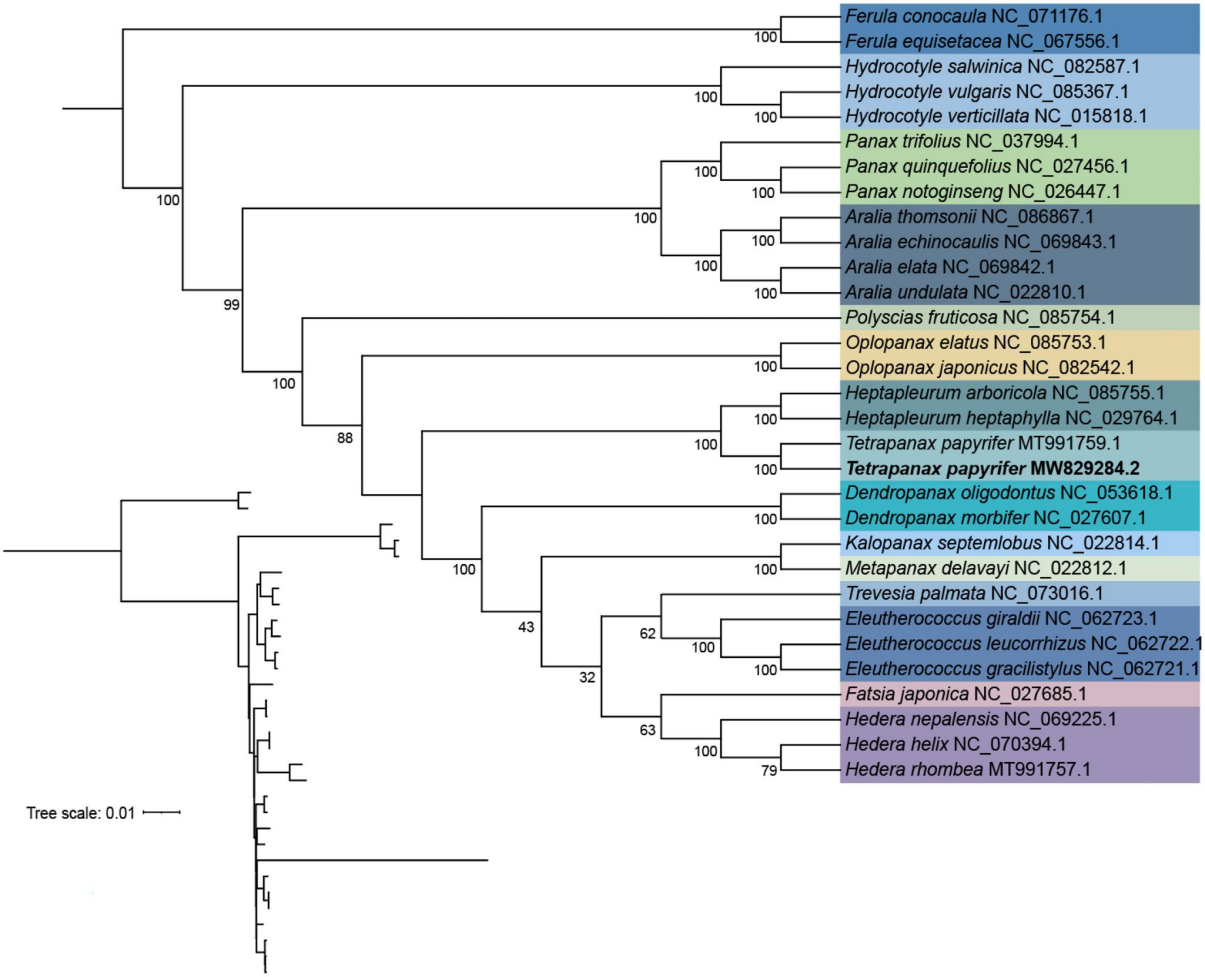
The phylogenetic tree was constructed using maximum-likelihood method based on complete chloroplast genome sequences. *Ferula conocaula* (NC_071176.1) (Qin et al. 2023) and *Ferula equisetacea* (NC_067556.1) (Yang et al. 2022) were used as outgroups in the phylogenetic tree. Species sequenced in this study are indicated in bold, and the accession numbers of species used for phylogenetic analysis are as follows: *Tetrapanax papyrifer* (MT991759.1), *Aralia undulata* (NC_022810.1), *Aralia elata* (NC_069842.1), *Aralia echinocaulis* (NC_069843.1), *Aralia thomsonii* (NC_086867.1), *Eleutherococcus giraldii* (NC_062723.1), *Eleutherococcus leucorrhizus* (NC_062722.1), *Eleutherococcus gracilistylus* (NC_062721.1), *Hedera helix* (NC_070394.1), *Hedera nepalensis* (NC_069225.1), *Hedera rhombea* (MT991757.1), *Hydrocotyle verticillata* (NC_015818.1) (Downie and Jansen 2015), *Hydrocotyle vulgaris* (NC_085367.1) (Luo et al. 2024), *Hydrocotyle salwinica* (NC_082587.1), *Panax notoginseng* (NC_026447.1) (Dong et al. 2014), *Panax quinquefolius* (NC_027456.1) (Kim et al. 2015), *Panax trifolius* (NC_037994.1), *Fatsia japonica* (NC_027685.1) (Chen et al. 2016), *Kalopanax septemlobus* (NC_022814.1) (Vasyutkina and Adrianova 2021), *Oplopanax japonicus* (NC_082542.1), *Oplopanax elatus* (NC_085753.1), *Trevesia palmata* (NC_073016.1), *Heptapleurum arboricola* (NC_085755.1), *Heptapleurum heptaphyllum* (NC_029764.1), *Metapanax delavayi* (NC_022812.1), *Polyscias fruticosa* (NC_085754.1), *Dendropanax oligodontus* (NC_053618.1) (Wang et al. 2022), and *Dendropanax morbifera* (NC_027607.1) (Kim et al. 2016).

Chloroplast genome sequences of 28 previously reported Araliaceae species were downloaded from the NCBI database, along with two Apiaceae species *Ferula conocaula* (NC_071176.1) and *Ferula equisetacea* (NC_067556.1) used as outgroups (Figure 3). Multiple sequence alignment was performed using MAFFT v 7.505 (Katoh and Standley 2013). A maximum-likelihood (ML) tree was constructed using IQ-TREE v 3.0.1 (Lanfear et al. 2020) under the K3Pu + F + R4 model with 1000 bootstrap replicates. Use iTOL (Interactive Tree of Life) (Letunic and Bork 2024) online software for visualization and beautification (Figure 3).

## Results

Approximately 10.9 Gb of raw data of *T. papyrifer* was obtained. The complete chloroplast genome of *T. papyrifer* spans 156,165 base pairs (bp). The coverage depth ranges from a minimum of 342× to a maximum of 22,817× (Figure S1). The chloroplast genome had a typical quadripartite structure, consisting of a large single copy (86,237 bp), a small single copy (17,956 bp), and two inverted repeat regions (25,986 bp). The chloroplast genome encodes 131 functional genes, comprising 87 protein-coding genes, 36 tRNA genes, and eight rRNA genes. Analysis of intron–exon structures revealed that 11 protein-coding genes (*rps16*, *atpF*, *rpoC1*, *petB*, *petD*, *rpl16*, *rpl2* × 2, *ndhB* × 2, *ndhA*) each contain one intron (Figure S2), while two genes (*clpP* and *pafI*) contain two introns (Figure S3).

According to the ML tree, *T. papyrifer* clustered with *Heptapleurum arboricola* (NC_085755.1) and *H. heptaphyllum* (NC_029764.1) to form a strongly supported clade (bootstrap = 100), indicating that *T. papyrifer* is most closely related to *Heptapleurum* among the taxa sampled.

## Discussion and conclusions

In this study, we sequenced and assembled the complete chloroplast genome of *T. papyrifer*. Compared with the previously published genome available in NCBI (MT991759.1), our assembly was 141 bp longer. IR boundary comparisons (Figure S4) showed that, except for the SSC region, the other three regions were slightly longer in our assembly. The *ndhF* and *rps19* genes had identical lengths in both genomes, whereas the *ycf1* gene differed by 12 bp. Overall, the two genomes exhibited only minor differences, indicating that the chloroplast genome structure of *T. papyrifer* is highly conserved.

Regarding genome annotation, several improvements were made compared with the previously published chloroplast genome of *T. papyrifer* (MT991759.1). In that genome, the *ycf15* gene was annotated with GTG as the start codon, whereas in our assembly (MW829284.2), the annotation was corrected and ATG was identified as the start codon. In addition, the functional gene *accD*, which was not annotated in the previously published genome (MT991759.1), was confirmed and annotated in this study. These refinements improve the accuracy and completeness of the chloroplast genome annotation of *T. papyrifer*.

The chloroplast genome of *T. papyrifer* is 156,165 bp in length, and its overall structure and size are comparable to those of other species within the Araliaceae family (Ge et al. 2017; Dong et al. 2022), most of which range between 150 and 160 kb in total length. *Tetrapanax* has been reaffirmed as a monotypic genus (Nesbitt et al. 2010; Li and Wen 2016), and its chloroplast genome characteristics support its distinct phylogenetic position within the order Apiales. To clarify its placement and significance within this family, we reconstructed a phylogenetic tree using the ML method based on complete chloroplast genome sequences. The results showed that *T. papyrifer* exhibits the closest phylogenetic relationship with *Heptapleurum*.

In summary, the complete chloroplast genome of *T. papyrifer* provides valuable insights into the phylogenetic relationships among species within the Araliaceae family and serves as an important reference for future comparative genomic and evolutionary studies across the order Apiales. The refined genome annotation presented in this study enhances the accuracy and completeness of the chloroplast genome resource of *T. papyrifer*.

## Supplementary Material

Supplemental Material

## Data Availability

The genome sequence data that support the findings of this study are openly available in GenBank of NCBI at https://www.ncbi.nlm.nih.gov/ under the accession number MW829284.2. The associated BioProject, SRA, and Bio-Sample numbers are PRJNA715070, SRR13985462, and SAMN18325377, respectively.
