# Supplementary material for: Chloroplast genome sequencing and phylogenetic analysis of Tetrapanax papyrifer (Hook.) K. Koch (Araliaceae)
Source: Mitochondrial DNA B Resour. 2026 Feb 3;11(3):362–6. doi: 10.1080/23802359.2026.2621456 (PMC12872081; doi:10.1080/23802359.2026.2621456)
Supplement: Supplemental Material [file TMDN_A_2621456_SM9231.docx]

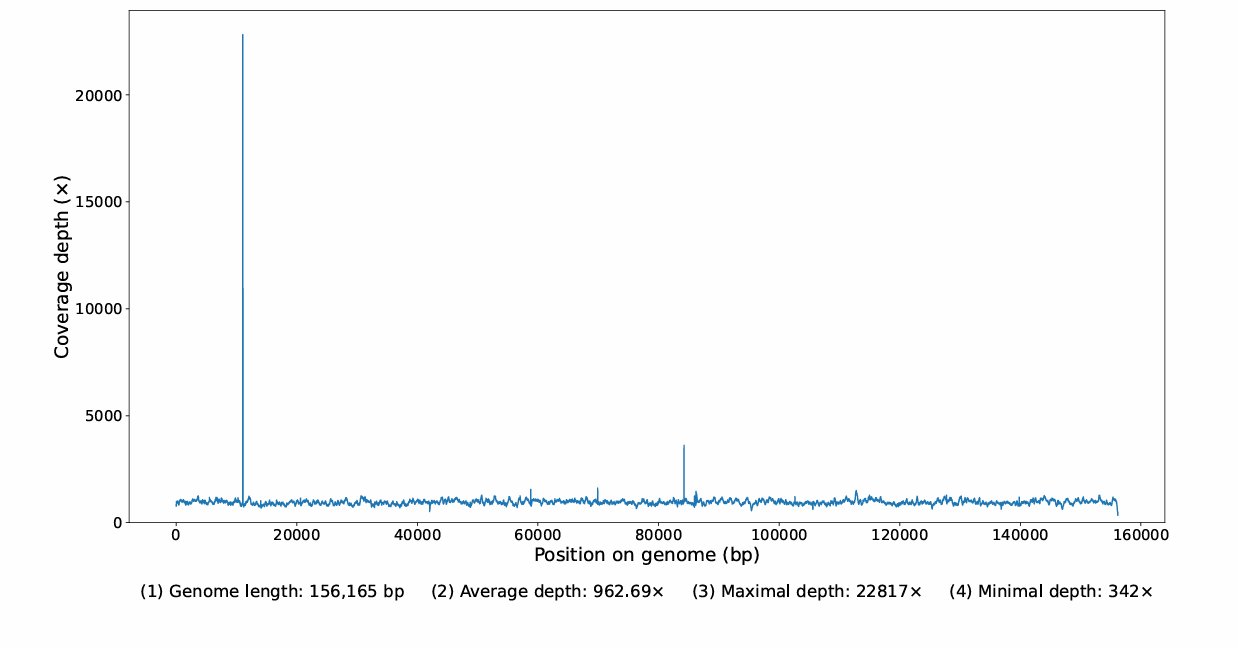


Figure S1. Illustrate the distribution characteristics of sequencing depth (Y-axis) along genomic positions (X-axis) in *Tetrapanax papyrifer*.


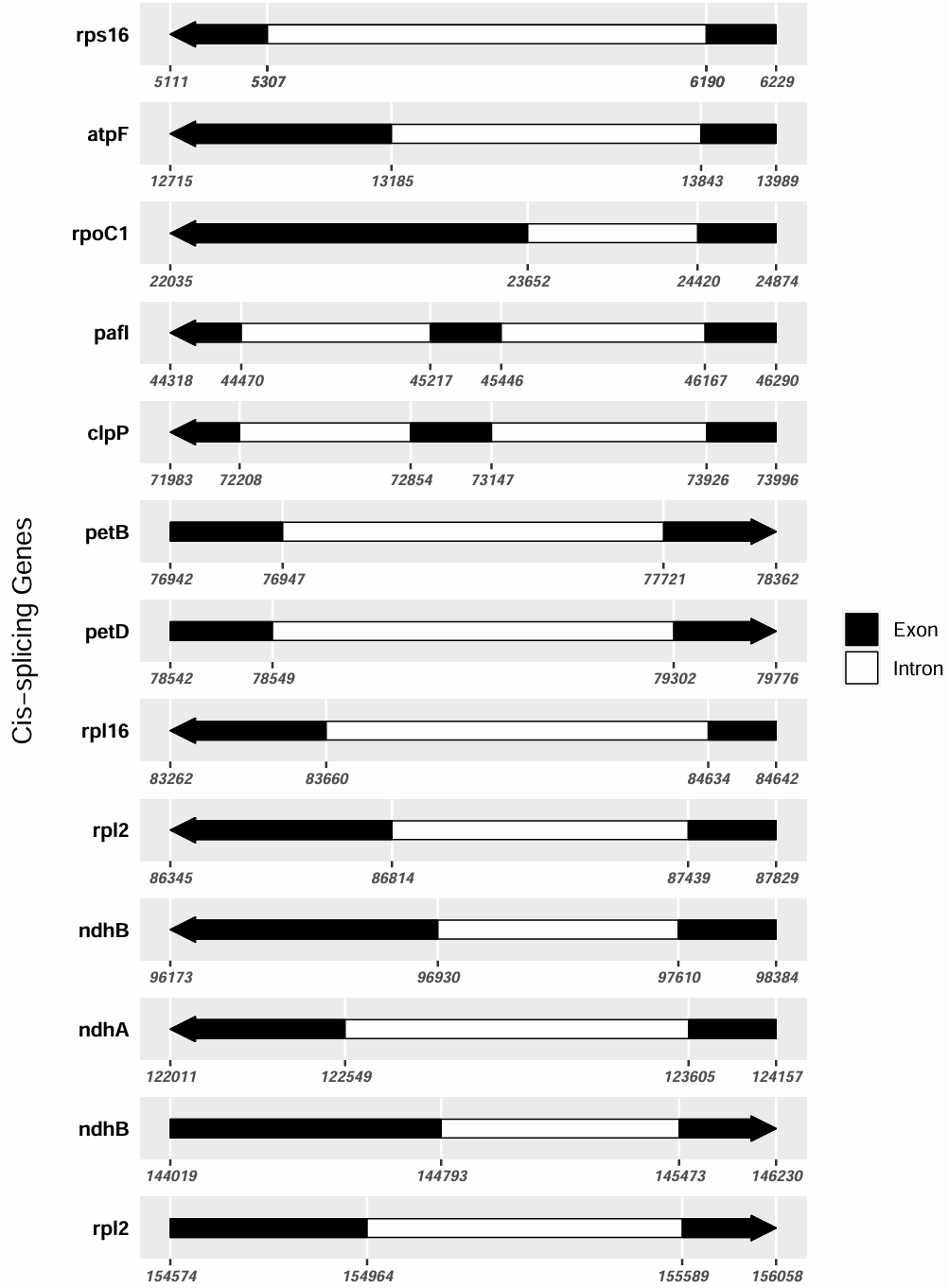


Figure S2. The map illustrates the distribution of cis-splicing genes within the chloroplast genome of *Tetrapanax papyrifer*. The direction of each gene is indicated by an associated arrow. Each gene structure comprises exons (represented by black segments) and introns (depicted as white segments), with gene names labeled on the left side of the map. The numbers beneath each arrow denote the corresponding gene's position within the genome.


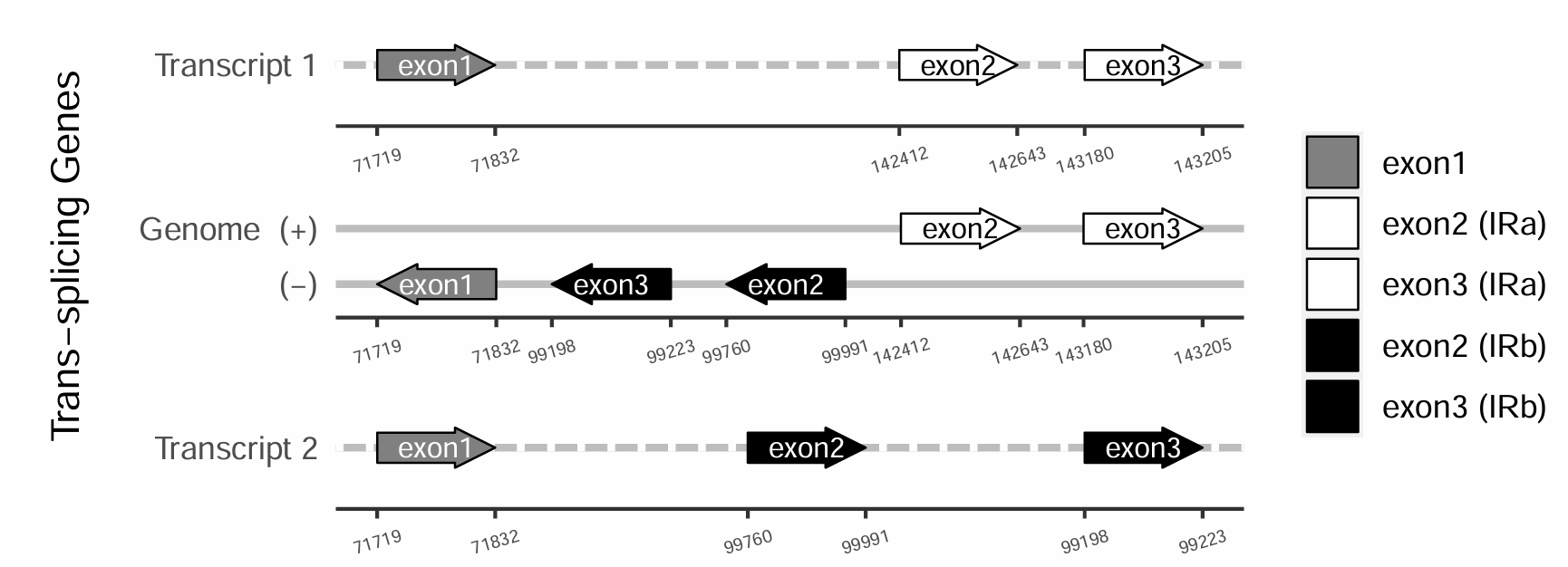


Figure S3. Schematic representation of the trans-splicing gene *(rps12*) within the chloroplast genome of *Tetrapanax papyrifer*.


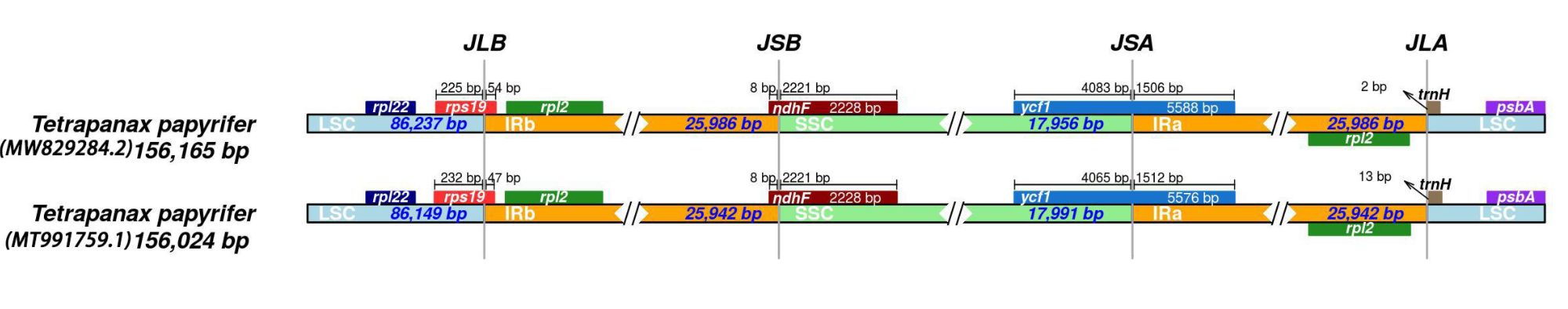


Figure S4. Comparison of boundary positions between the single-copy regions (large, LSC; small, SSC) and the inverted repeat (IR) regions in the newly assembled chloroplast genome (MW829284.2) and the previously published genome (MT991759.1). The positions of genes located at the IRb/LSC, IRb/SSC, IRa/SSC, and IRa/LSC junctions are shown, with distances from the respective boundaries indicated.
